# Impact of ^18^F-FDG-PET/CT on Clinical Management in Patients with Cholangiocellular Carcinoma

**DOI:** 10.1259/bjro.20210008

**Published:** 2021-07-05

**Authors:** Lena Sophie Kiefer, Julia Sekler, Brigitte Gückel, Mareen Sarah Kraus, Christian la Fougère, Konstantin Nikolaou, Michael Bitzer, Sergios Gatidis, Christina Pfannenberg

**Affiliations:** 1 Department of Radiology, Diagnostic and Interventional Radiology, University Hospital Tuebingen, Hoppe-Seyler-Strasse 3, 72076 Tuebingen, Germany; 2 Department of Radiology, Nuclear Medicine, University Hospital Tuebingen, Hoppe-Seyler-Strasse 3, 72076 Tuebingen, Germany; 3 Department of Gastroenterology, Gastrointestinal Oncology, Hepatology and Infectious Diseases, University Hospital Tuebingen, Otfried-Müller-Strasse 10, 72076 Tuebingen, Germany

## Abstract

**Objective:**

To determine the impact of ^18^F-FDG-PET/CT on clinical management of patients with cholangiocellular carcinoma (CCA).

**Methods:**

Patients with CCA undergoing clinically indicated ^18^F-FDG-PET/CT between 04/2013 and 08/2018 were prospectively included in a local PET/CT registry study. Intended clinical management (“non-treatment” such as watchful-waiting or additional diagnostic tests, and “palliative” or “curative treatment”) was recorded before and after PET/CT. Changes in intended management after PET/CT were analyzed.

**Results:**

27 patients (mean age: 60 years, IQR: 51.5–67.5 years, 56% males) with 43 PET/CT examinations were included. Intended management changed in 35/43 cases (81.4%) following PET/CT. Major changes (*i.e.,* between “non-treatment” and “treatment” strategies or between a “curative” and “palliative” treatment goal) occurred in 27/43 (62.8%) cases. Before PET/CT, additional imaging and/or biopsy were intended in 21/43 (48.8%) and 9/43 (20.9%) cases, respectively. After PET/CT, further imaging was carried out in one case and imaging-targeted biopsy in eight cases. Although the absolute number of biopsies after PET/CT did not decrease, in only one of these eight cases biopsy had already been planned before PET/CT, whereas in the other eight cases, the originally planned biopsies were dispensable after PET/CT.

**Conclusions:**

^18^F-FDG-PET/CT significantly impacts clinical management of patients with CCA. It guides decisions on treatment strategy (especially curative *vs* palliative treatment goal) and on additional tests, particularly by helping referring clinicians to avoid unnecessary imaging and by guiding targeted biopsy.

**Advances in knowledge:**

Systematic implementation of ^18^F-FDG-PET/CT may enable a more appropriate and tailored treatment of patients with CCA, especially in cases of suspected recurrence.

## Introduction

Cholangiocellular carcinoma (CCA) is a rare malignant neoplasm originating from the biliary duct epithelium with an aggressive behavior and a poor prognosis. It is the second most common primary hepatobiliary tumor (—10–15% of primary liver cancers) with an increasing incidence over the past years (currently 0.3–3.5/100 000 in most Western countries).^1–4^ Based on the location of the primary tumor, CCAs are subclassified into intrahepatic (iCCA) and extrahepatic CCAs (eCCA), the latter including perihilar (Klatskin tumor) and distal bile duct cancer.^5^

Both primary and recurrent CCA frequently remain symptomatically indolent or present with nonspecific symptoms until advanced tumor stages. Therefore, CCA poses a diagnostic and therapeutic challenge. Treatment options depend on tumor size, location, invasion of adjacent structures, extent of bile duct involvement, and distant metastatic disease. Currently, radical surgical resection with clear margins is considered the only potential curative treatment option for CCA patients.^6,7^ The introduction of multidisciplinary treatment strategies including postoperative adjuvant chemo- and/or radiation therapy as well as aggressive treatment in case of recurrence has slightly improved the prognosis.^8,9^ Still, the postoperative (local and distant) recurrence rate is up to 60% and the 5-year overall survival rate after surgery is still only 15–40%.^7,10^ These numbers further emphasize the aggressive nature of CCA and the importance of early detection and accurate staging as well as patient allocation to an appropriate treatment regimen.

Imaging plays an important role in diagnosing and staging of both primary and recurrent CCA, as well as in assessing therapy response. The routine clinical workup regarding imaging mainly includes magnetic resonance imaging (MRI) and contrast-enhanced computed tomography (CECT).^6^ Although 2-deoxy-2-^18^F-fluoro-D-glucose positron emission tomography/computer tomography (^18^F-FDG-PET/CT) has gained in importance recently, its role in CCA is still controversial and recommended only on a case-by-case basis.^6^ Besides emerging evidence for the superior diagnostic performance of ^18^F-FDG-PET/CT compared to that of CT/MRI alone, particularly in patients with recurrent CCA^11,12^, it remains unclear whether PET/CT has a relevant impact on clinical decision-making, specifically with regard to changes in patient management and associated survival.

Therefore, the aim of our study was to determine the impact of ^18^F-FDG- PET/CT on clinical management of CCA patients. Our hypothesis was that ^18^F-FDG- PET/CT findings substantially influence clinical decision-making regarding treatment allocation and help referring physicians to avoid unnecessary diagnostic tests.

## Methods and materials

### Registry study

This study was based on the database of a local PET/CT registry that includes a total of 5,508 patients (status 08/2018).^13^ The study was approved by the University Institutional Review Board and the local ethics committee. Written informed consent was obtained from all patients.

The design of the registry-study has been described in detail previously.^13^ This single-center registry comprises a prospective cohort of 5,508 patients who received a clinically indicated PET/CT examination. Comprehensive and standardized questionnaire data from the referring physicians were prospectively collected to determine the impact of PET/CT on patient management, clinical decision-making and intended use of diagnostic procedures.

### Study cohort

The study cohort comprises all CCA patients with that were prospectively included in the local PET/CT registry from 04/2013 to 08/2018. In case of patients with repeated PET/CTs, also consecutive examinations were included. Pre-PET/CT imaging was obtained from patient records and the mean time interval between preceding imaging and PET/CT examination was recorded. Results of preceding and subsequent histopathological examinations were documented. Patient records were used to review consistency of questionnaire information and documented clinical management procedures as well as for documentation of patient outcome.

### Questionnaire data

Referring physicians completed standardized questionnaires before and after PET/CT. The design of the questionnaires followed the conception of the "National Oncologic PET Registry” (NOPR) and has been described in details previously.^13,14^ In brief, the pre-PET/CT questionnaire contained items on PET/CT indication (“diagnosis”, “staging”, and “suspected recurrence”) and intended clinical management (“curative treatment”, “palliative treatment”, and “non-treatment” such as additional diagnostic tests or watchful waiting). The post-PET/CT questionnaire contained the same items on intended management based on the knowledge of the PET/CT results and further items on change of oncological status.

### Definition of changes in intended management

**Figure 1.**
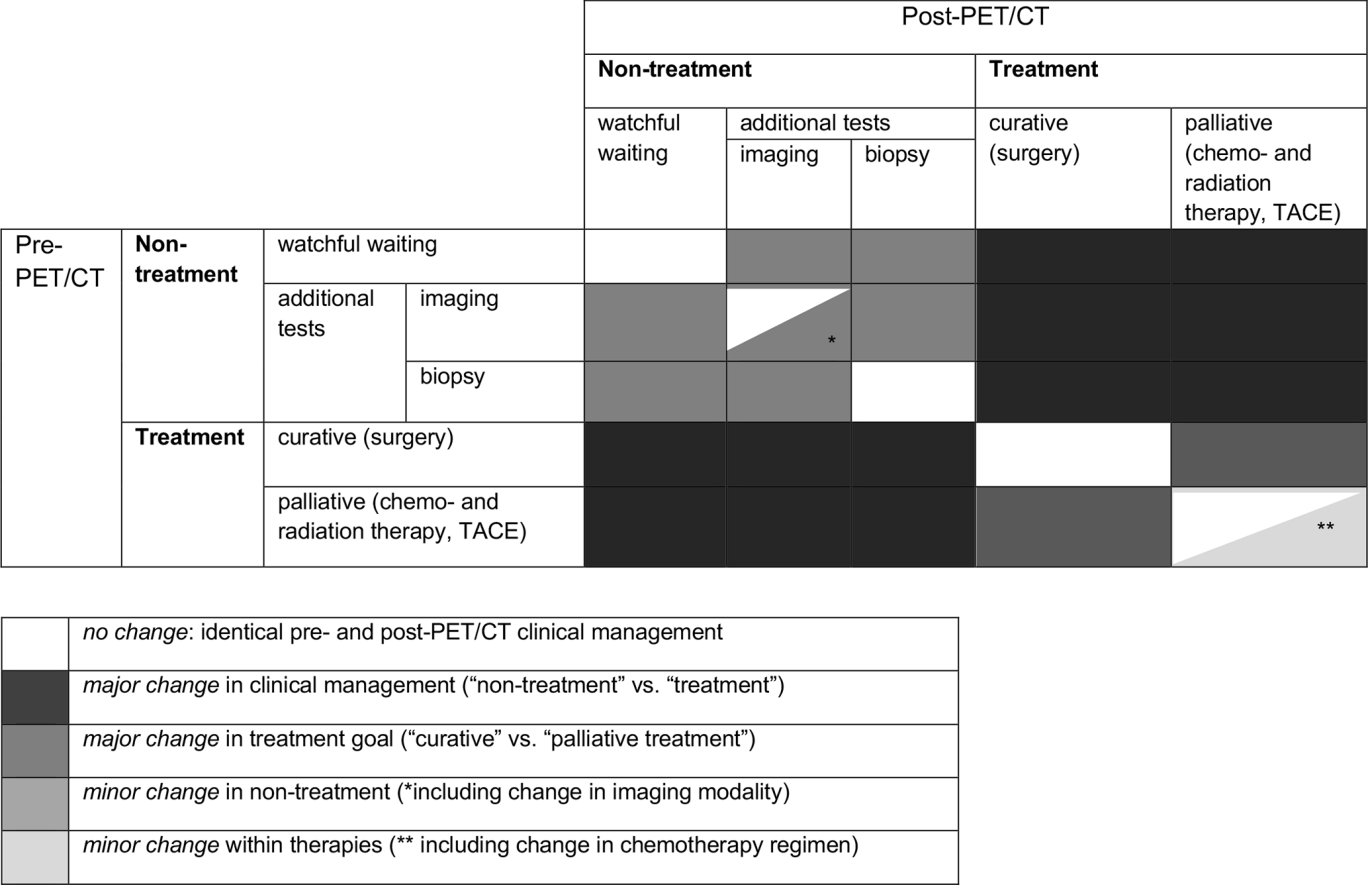
Definition of changes in clinical management in patients with CCA.

Changes in intended management in CCA patients were assessed on the basis of two different approaches.^14^ The first, more general approach dichotomizes the intended management strategy as either “treatment” (surgery, chemo-/radiation therapy, TACE, and combinations) or “non-treatment” (watchful waiting and additional diagnostic tests, such as imaging and/or biopsy) with changes as a consequence of PET/CT occurring between those two groups. The second, more specific approach, also considers the treatment goal, classifying it into “curative” (surgical resection/liver transplantation) or “palliative” (systemic chemotherapy including targeted therapy, loco-regional therapy such as radiation therapy and TACE, and combinations) (Figure 1).

A *major change* in management was defined as a change in either clinical management (“non-treatment” *vs* “treatment”) or treatment goal (“curative” *vs* “palliative”). In contrast, a *minor change* was defined as a change within the “nontreatment” category (such as changes in additional tests) or as a change within the treatment category (*e.g.,* adjustments in chemotherapy regimen/irradiation field) without changing the categorical treatment goal.

### ^18^F-FDG-PET/CT Examination

PET/CT examinations were performed on a Biograph mCT (Siemens Healthineers, Knoxville, TN). Patients fasted for at least 12 h before the examination and had blood glucose levels < 150 mg dl^−1^ at the time of ^18^F-FDG- injection. Image acquisition was started 60 min after i.v. application of 300–350 MBq ^18^F-FDG. Examinations were performed in supine position with elevated arms and with patients being embedded in a vacuum mattress. In the absence of contraindications, CT images were obtained in portal venous phase 80 s after weight-adapted i.v. injection of 80–140 ml Ultravist370 (Schering AG, Berlin, Germany) at 1.0–2.5 ml s^−1^ followed by a 40-ml saline flush. PET-images were acquired over 6–8 bed positions (acquisition time: 2 min/bed position) covering an area from the skull base to the mid-thigh and reconstructed using a 3D-ordered subset expectation maximization algorithm (2 iterations, 21 subsets, Gaussian filter 2.0 mm, matrix size 400 × 400, slice thickness 2.0 mm). All PET/CT examinations were read in an interdisciplinary setting by experienced radiologists and specialists for nuclear medicine.

### Statistical analysis

Demographics and characteristics of the study population are presented as median with 1st and 3rd quartile (interquartile range: IQR) for continuous variables and absolute counts with percentages for categorical variables. Changes in intended management were assessed as proportions with the 95% confidence intervals (95% CI) of these proportions being calculated using the normal approximation for a binomial distribution. For the analysis of changes in management, all PET/CT examinations including repeated PET/CTs were considered. Kaplan–Meier analysis was performed to determine differences of overall estimated survival between different treatment groups. For the analysis of survival, only the first PET/CT statistical performed examination was considered in cases of repeated examinations. Significance was indicated by p-values <0.05. Statistical analysis was using SPSS version 24 (IBM Corporation, Armonk, North Castle, NY).

## Results

### Study cohort

27 patients with CCA (mean age: 60.0 years, IQR: 51.5–67.5 years, 56% males) with 43 ^18^F-FDG-PET/CT examinations in total between 04/2013 and 08/2018 were identified from the PET/CT registry database and included in this study. Based on the localization of the primary tumor, 15/27 patients (55.6%) were subcategorized with an iCCA and 12/27 patients (44.4%) with an eCCA. Detailed patient demographics and characteristics are provided in Table 1.

**Table 1.** Patient demographics and characteristics

	*Number of patients (N = 27)*	*%*
**Gender**		
female	12	44.4
male	15	55.6
**Age at PET/CT (in years**)		
mean	60.0	
median	62.0	
interquartile range (first and third Quartile)	51.5–67.5	
**In-/outpatients**		
inpatients	24	88.9
outpatients	3	11.1
Last preceding imaging before PET/CT* (*of *N* = 43)		
CT	17	39.5
MRI	17	39.5
PET/CT	7	16.3
not documented	2	4.7
**Time interval between preceding imaging and PET/CT (in days)**		
mean	63.6	
median	34.0	
interquartile range (first and third Quartile)	15.0–83.0	
**Number of PET/CT scans per patient**		
one	14	51.9
two	10	37.0
three	3	11.1
**CCA-Subtypes based on location**		
iCCA	15	55.6
eCCA	12	44.4
hilar (Klatskin tumor)	7	
distal bile duct	5	

CCA: Cholangiocarcinoma

Preceding imaging before PET/CT was mainly cross-sectional imaging by CT (39.5%) or MRI (39.5%). In 7/43 cases (16.3%), two consecutive PET/CT examinations were documented and in 2/43 cases (4.7%) no preceding imaging was documented. The mean time interval between the preceding imaging and the PET/CT examination was 63.6 days (IQR: 15.0–83.0 days). 16/27 patients received only one PET/CT examination, 10/27 patients received two and 3/27 patients received three PET/CTs.

### PET/CT Indication

Data on PET/CT indications are provided in Table 2. In 2/43 cases (4.7%), PET/CT was performed for “diagnosis” without any preceding biopsy. In both of these cases, the referring clinicians suspected a different tumor entity. In 41/43 cases (95.3%), prior histopathology had already confirmed CCA. In 46.5%, PET/CT indication was “staging”, with two cases being performed for “primary staging” and 18 cases being performed for “re-staging” after therapy/during interval monitoring. In 48.8%, PET/CT was performed because of suspected recurrence, with a new lesion detected in preceding imaging in 17 cases and clinically suspected recurrence (new jaundice, altered laboratory markers (parameters of cholestasis), and/or elevated tumor markers (CA 19–9 and CEA)) in 10 cases.

**Table 2.** PET/CT indications

	*Number of PET-CT scans (N = 43*)	*%*
Indication for PET/CT^a^		
diagnosis (without prior biopsy)	2	4.7
staging	20	46.5
primary staging	2	
re-staging after therapy^b^	18	
interval monitoring	3	
residual tumor & vitality	5	
therapy response	11	
suspected recurrence^c^ ^d^	21	48.8
new lesion in preceding imaging	17	
laboratory & tumor markers	10	

CCA, Cholangiocarcinoma.

a PET/CT indications: diagnosis (suspected primary), staging (primary staging and re-staging after therapy of histologically confirmed cancer), and suspected recurrence of previously treated cancer

b of 18 cases re-staging after last documented therapy: 13 cases during/after chemotherapy, two cases after (re-) surgery, two cases after TACE, and one case after radiation therapy

c of 21 cases with suspected recurrence (last documented therapy): 19 cases after chemotherapy, one case after re-surgery, and one case after radiation therapy

d multiple selection possible

### PET/CT Findings

In 25/43 examinations (58.1%), PET/CT detected new lesions suspicious for metastases, and these lesions were either not detected or not interpreted as metastases in preceding conventional imaging. Most frequently, CCA metastases were found in the liver (22/43 cases), lymph nodes (13/43 cases), peritoneum (11/43 cases), and retroperitoneum/abdominal wall (8/43 cases).

Regarding the oncological status, PET/CT led to an upgrade in the setting of known malignancy in 24/43 cases (55.8%), thereby mainly proving prior unsuspected, distant metastatic disease. Furthermore, PET/CT confirmed suspected local recurrence in 10/43 cases (23.3%). A downgrade of the oncological status was documented in 1/43 cases (2.3%), whereas in 8/43 cases (18.6%), no change of oncological status occurred and/or no recurrence was found.

### Changes in intended management after PET/CT

**Figure 2.**
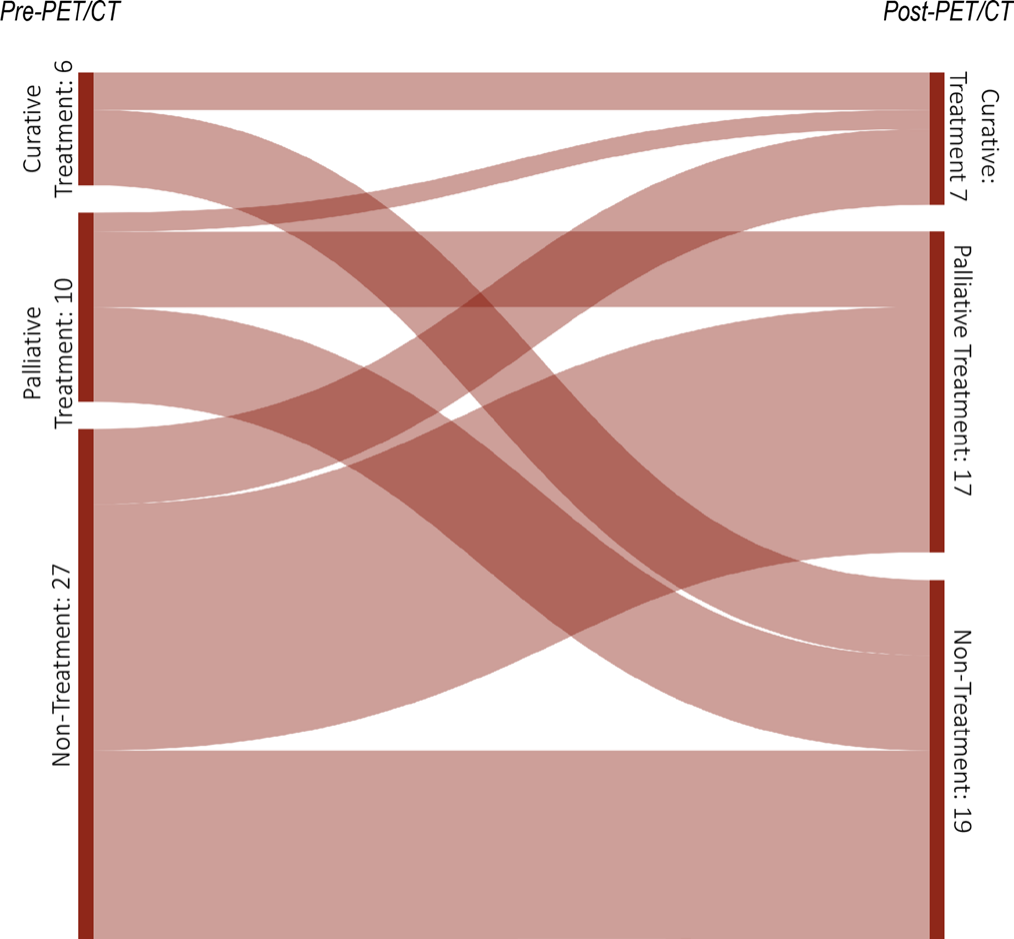
Sankey diagram showing the frequency of changes in the “treatment” and “non-treatment” management category under consideration of the treatment goal after ^18^F-FDG-PET/CT in 27 patients with CCA. The widths of the bands are directly proportional to the number of PET/CT scans (*N* = 43).

**Figure 3.**
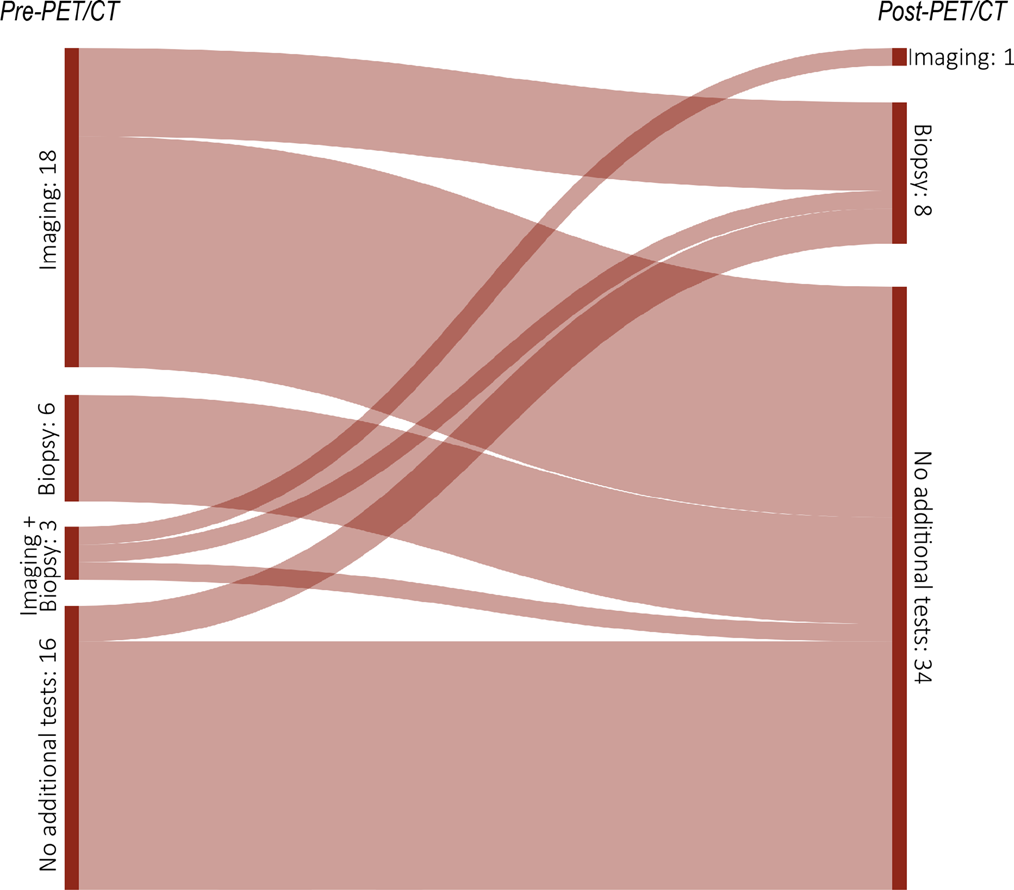
Sankey diagram showing the frequency of changes in the demand for additional tests (imaging and/or biopsy) after ^18^F-FDG-PET/CT in 27 patients with CCA. The widths of the bands are directly proportional to the number of PET/CT scans (*N* = 43).

The impact of PET/CT on intended clinical management in CCA patients is presented in (Tables 3 and 4) and illustrated in (Figures 2 and 3) . Overall, major and minor changes in intended management were observed in 35/43 cases (81.4%) based on the results of PET/CT.

**Table 3.** Impact of ^18^F-FDG-PET/CT on intended clinical management stratified generally as “treatment” *vs* “non-treatment” (*N* = 43)

			Indication for PET/CT			All Patients
Management plan	Pre-PET/CT	Post-PET/CT	Diagnosis	Staging	Recurrence	
No. of scans per indication (%)			2 (4.7%)	20 (46.5%)	21 (48.8%)	43 (100%)
	Treatment^a^	Treatment^a^	-	6 (14%)	1 (2.3%)	7 (16.3%)
	Non-treatment^b^	Non-treatment^b^	-	3 (7.0%)	7 (16.3%)	10 (23.3%)
	Non-treatment^b^	Treatment^a^	-	10 (23.3%)	7 (16.3%)	17 (39.5%)
	Treatment^a^	Non-treatment^b^	2 (4.7%)	1 (2.3%)	6 (14.0%)	9 (20.9%)
Change in clinical patient management			2 (100%)	11 (55%)	13 (61.9%)	26 (60.5%)
95% confidence interval				32.0–78.0%	40.5–83.3%	45.5–75.5%

a Treatment: surgical resection, liver transplantation, systemic chemotherapy, TACE, radiation therapy, and combinations.

b Non-treatment: watchful waiting, and additional diagnostic tests (*e.g.,* imaging, biopsy).

**Table 4.** Impact of ^18^F-FDG-PET/CT on intended clinical management stratified by treatment goal (“curative” and “palliative treatment”) (*N* = 43)

Management plan		Change in management*	No. of scans (%)
Pre-PET/CT	Post-PET/CT		
Curative	Curative	No change^a^	2 (4.7%)
	Palliative	Major change^b^	-
	Non-treatment	Major change^c^	4 (9.3%)
Palliative	Curative	Major change^b^	1 (2.3%)
	Palliative	No change^a^ Minor change^d^	3 (7.0%) 1 (2.3%)
	Non-treatment	Major change^c^	5 (11.6%)
Non-treatment	Curative	Major change^c^	4 (9.3%)
	Palliative	Major change^c^	13 (30.2%)
	Non-treatment	No change^a^ Minor change^e^	3 (7.0%) 7 (16.3%)
Overall change in clinical management			35 (81.4%)
Major change^1 & 2^			27
Minor change^3 & 4^			8
*No change* in patient management^a^			8 (18.6%)

a no change: identical pre- and post-PET/CT clinical management

b major change in therapy goal (“curative” *vs* “palliative treatment”)

c major change in clinical management (“non-treatment” *vs* “treatment”)

d minor change among therapies (** including change in chemotherapy regimen)

e minor change in non-treatment (*including change in imaging test)

f based on definitions of no change, minor and major change in clinical management in patients with CCA (also see Figure 1):

*Major changes* between a “treatment” (surgery, chemo-/radiation therapy, TACE) and a “non-treatment” (watchful waiting/additional diagnostic tests) strategy occurred in 26/43 cases after PET/CT (60.5%, 95% CI: 45.5–75.5%). In detail, a “non-treatment” strategy changed to “treatment” in 17/43 cases (39.5%), whereas a “treatment” strategy changed to “non-treatment” in 9/43 cases (20.9%). Hence, a change from a “non-treatment” strategy to “treatment” was substantially more likely than the opposite change. Thereby, most changes occurred if indication for PET/CT was “suspected recurrence” (13/43 cases: 61.9%). If the treatment goal (“curative” *vs* “palliative”) is also considered, referring physicians revised their “non-treatment” strategy before PET/CT into an intended “curative” treatment goal in 4/43 cases (9.3%) and into an intended “palliative” treatment goal in 13/43 cases (30.2%). In all of these four cases with a change to an intended “curative” treatment goal, PET/CT proved limited tumor extent of recurrent disease and ruled out distant metastatic disease, thus allowing for a curative surgical approach. In contrast, an intended “curative” or “palliative treatment” goal before PET/CT was revised into a “nontreatment” strategy in 9/43 cases (20.9%). In 17/43 cases (39.5%), *no change* in clinical management was documented after PET/CT. Thereby, a “treatment” plan remained unchanged in 7/43 cases (16.3%) and a “non-treatment” strategy remained the same in 10/43 cases (23.3%).

*Minor changes* in the “non-treatment” category and among therapies without changes in treatment goal occurred in 8/43 cases (18.6%). Significant changes in demand for additional tests after PET/CT could be observed (Supplementary Table 1). Before PET/CT, referring physicians intended to perform additional tests in 30/43 cases (69.8%), mainly CECT and MRI. After PET/CT, imaging was performed in only 1/43 cases, with a multiphasic CECT examination being performed prior to hemihepatectomy. Biopsy was intended in 9/43 (20.9%) cases before PET/CT and was performed in 8/43 (18.6%) cases after PET/CT. Yet, in only one case, the pre-PET/CT planned biopsy was actually carried out afterwards. In all the other eight cases, the originally planned biopsies were dispensable after PET/CT. However, the results of the PET/CT lead to new image-guided biopsies in eight patients because of reported suspicious sites or needed histopathological proof for newly detected metastatic disease. Out of these eight cases with PET/CT-guided biopsy, histopathological examination proved malignancy in seven cases. Overall, PET/CT results prevented additional tests in 62.8% (27/43 cases), with prevention of imaging in 19/43 cases, of biopsy in 6/34 cases and of both imaging and biopsy in two cases (Supplementary Table 1).

### Patient survival

**Figure 4.**
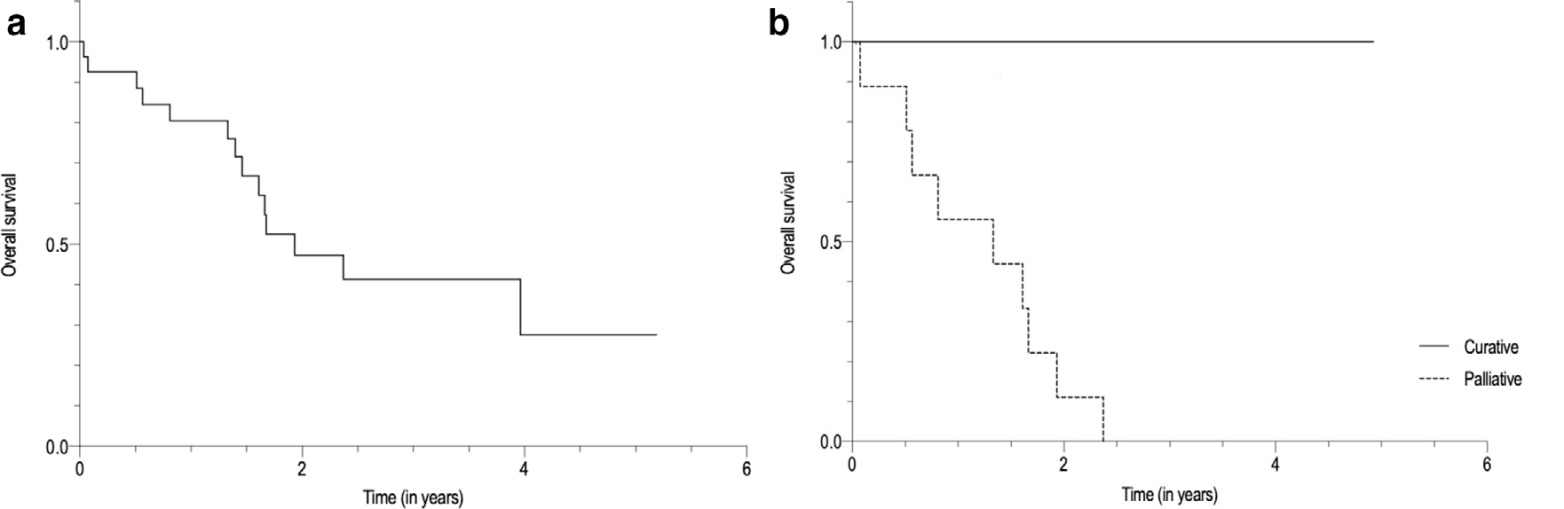
Survival of 27 patients with CCA after the first ^18^F-FDG-PET/CT examination. A Overall survival of all 27 patients (mean survival time after first ^18^F-FDG-PET/CT examination: 1.81 years (95% CI 1.29–2.34 years)). B Overall survival after first ^18^F-FDG-PET/CT examination of patients in whom a “curative treatment” regimen was intended after PET/CT (*N* = 6) (mean survival time: 2.21 years (95% CI 0.76–3.66 years), and in whom a “palliative treatment” regimen was intended after PET/CT (*N* = 12) (mean survival time: 1.21 years (95% CI 0.72–1.69 years).

Until the timepoint of this analysis, 14/27 patients (51.9%) had died. Estimated overall survival of all 27 patients after the first PET/CT examination was 1.81 years (95%-CI: 1.29–2.34 years). Survival curves are illustrated in Figure 4. In patients with intended "curative treatment” after PET/CT, estimated mean survival time was 2.21 years (95%-CI: 0.76-3.66 years), in comparison with an estimated mean survival time of 1.21 years (95%-CI: 0.72-1.69 years) in patients with a "palliative” treatment goal.

## Discussion

The objective of this study was to assess the impact of ^18^F-FDG-PET/CT on referring physicians’ plans of intended management in CCA patients. Our results indicate that ^18^F-FDG-PET/CT has a substantial impact on intended patient management, with a documented frequency of both major and minor changes in management plan of overall 81.4%. Thus, ^18^F-FDG-PET/CT may play an important role in the routine clinical workup and management of patients with CCA.

**Figure 5.**
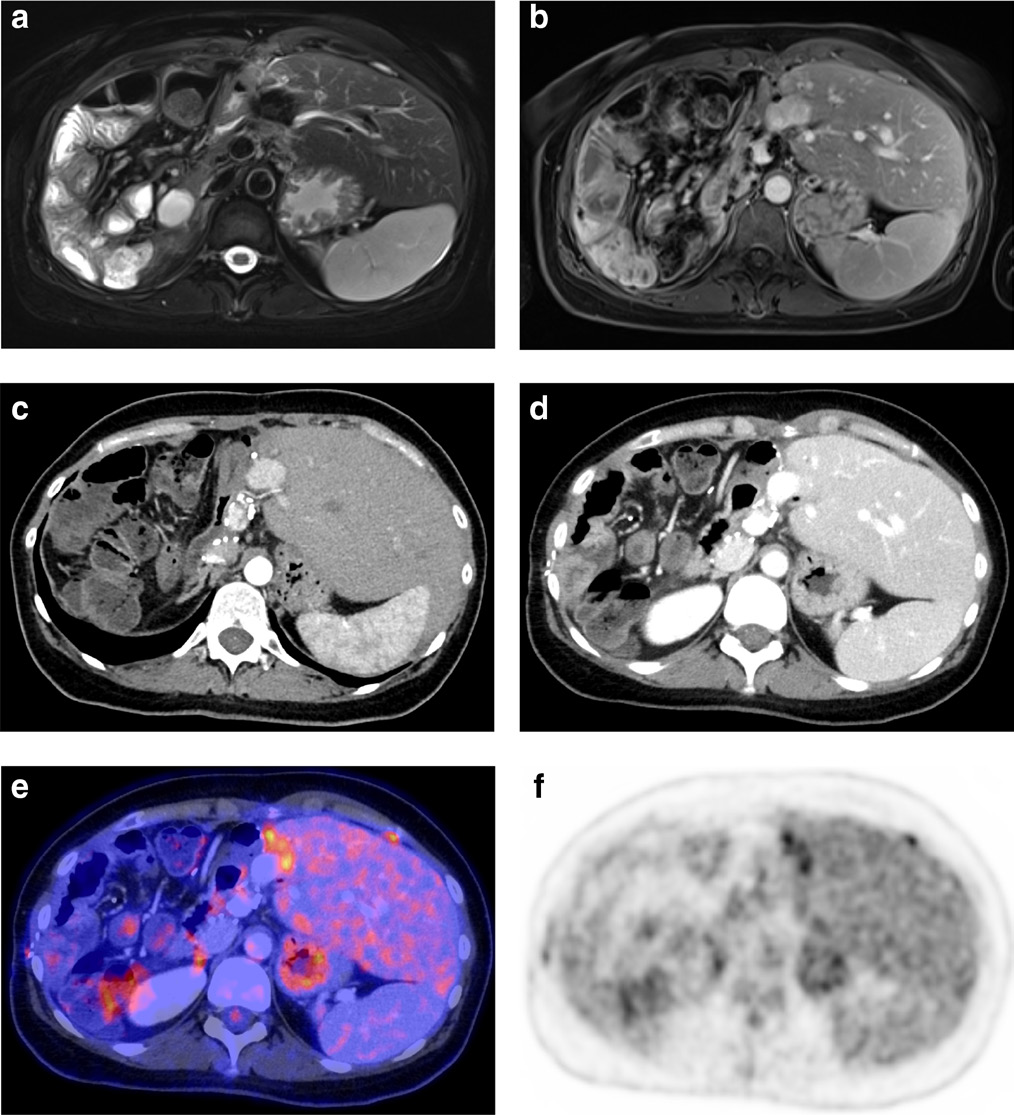
Example of a 36-year-old female patient with recurrent CCA in the left liver lobe after right hemihepatectomy and systemic chemotherapy (gemcitabine/cisplatin), not detected on CT and MRI. Recurrence was suspected clinically by elevated tumor markers (CA 19.9). Before PET/CT, clinicians intended further imaging and biopsy to prove suspected recurrence, and revised their “non-treatment” strategy into a “palliative treatment” goal (with change of chemotherapy regimen) after PET/CT (*major change*).

Data on PET/CT imaging of CCA is very limited, although it has been shown that all CCA-subtypes will be FDG-avid. The sensitivity and specificity of ^18^F-FDG- PET/CT for the evaluation of CCA varies by anatomic location and morphological variability of the primary tumor, being reported higher for iCCA (>90%) than for eCCA (~60%), whereas the detection rate of macroscopic distant metastatic disease approaches 100%.^1112^ Although current European and American guidelines acknowledge existing evidence for ^18^F-FDG-PET/CT, they still consider its role in the evaluation of CCA as “uncertain” and only recommend CECT and/or MRI in the routine clinical workup.^67^ Also, the amount of conducted clinical studies on PET/CT in CCA is very low, hardly providing reliable results. That explains why ^18^F-FDG-PET/CT has neither an established role in the CCA-diagnosis nor it is routinely recommended for CCA-staging. In this respect, our findings confirmed that ^18^F-FDG-PET/CT was not routinely requested for primary staging (only 4.7%) but appeared to play a more prominent role in the setting of re-staging during/after therapy and suspected recurrence (90.7%). In this context, an ambiguous clinical situation with newly detected lesions in other imaging modalities or altered laboratory and/or elevated tumor markers in absence of suspicious imaging findings in MRI/CT seemed to motivate the use of PET/CT (Figure 5). In such selected cases, referring clinicians have expected decisive findings regarding individual therapeutic decisions using PET/CT.

Due to the aggressive behavior and limited treatment options, identification and accurate determination of the extent of all primary and metastatic lesions are critical for patient management. Given that radical surgical resection with clear margins is still considered the only potential curative treatment option,^6,7^ precise staging is of major significance, both in the setting of timely diagnosis and primary staging, as well as in re-staging during/after therapy or in suspected recurrence. In this context, our results indicate that PET/CT seemed to be of particular clinical relevance to proof a limited tumor extent and to preclude distant metastatic disease, thus allowing for an aggressive surgical approach in a curative treatment strategy. In contrast, by demonstrating (previously unexpected) advanced disease stages, consecutively upgrading the oncological status, PET/CT allowed for an early allocation to a palliative treatment regimen, thus preventing patients from futile surgery.

Taken together, we found that clinical management of CCA patients from our registry study was revised in 81.4% after PET/CT. Until now, specific data on changes of clinical management following PET/CT in CCA has not been available. However, results from the NOPR demonstrated less frequent changes, occurring in 42.9% of patients with liver and intrahepatic bile duct cancer. Similarly to the NOPR, changes from a “non-treatment” to a “curative” and “palliative treatment” strategy were also substantially more likely in our cohort.^15^ These findings may generally reflect referring physicians’ assessment of a significantly different probability of extent of disease pre- *vs* post-PET/CT in CCA patients. This assumption may further be supported by the marked reduction in demand for further diagnostic tests following PET/CT. Based on the results of PET/CT, additional tests were only requested in a few individual cases with expected high clinical relevance or relevant add-on information (*e.g.,* arterial phase-CT prior hemihepatectomy), whereas in most cases, further (non-)invasive diagnostics became redundant after PET/CT. Specifically with respect to biopsies, PET/CT findings guided the referring clinicians to an optimal biopsy site in case of needed histopathological proof, rather than obviating biopsies entirely. However, by preventing additional tests in 62.8% of the cases in our study, PET/CT significantly reduced patient burdening and may thereby also have a relevant impact on patient’s quality of life.

Even though in this study ^18^F-FDG-PET/CT significantly changed intended management in CCA patients, it is still unclear whether such changes also have a significant impact on patient outcome and/or survival. In this cohort, mean survival time after the first PET/CT examination was longer in patients with an intended “curative treatment” regimen compared to those with an intended “palliative treatment” goal. Thus, PET/CT may generally facilitate correct patient allocation to a more effective treatment regimen by detecting and accurately staging potentially treatable metastases, which were previously not entirely identified by other imaging modalities. Ultimately, this may lead to a reduced treatment-associated morbidity and CCA-associated mortality as well as an improved overall outcome. Moreover, PET/CT also detected previously unsuspected and incurable metastatic disease, which frequently caused changes in intended management from a “curative” to a “palliative treatment” goal as described above or changes of chemotherapy regimen in cases of progressive disease. In these settings, PET/CT may not generally result in a reduction of CCA-mortality but may improve patients’ quality of life by avoiding ineffective treatment resulting in a lower rate of morbidity. However, changes in intended clinical management may, therefore, only be interpreted as surrogates for actual patient outcome.

General limitations of our local registry study and other, decision-impact studies based on observational data have been discussed in detail previously.^13,14^ Our registry study was designed in order to assess the self-reported changes in daily clinical management following PET/CT, whereas their implementations in clinical practice were not primarily incorporated. However, we found that the intended change in patient management derived from the questionnaires and documented procedures within patient records were reasonably consistent creating a sufficient clinical-based evidence. Although our academic hospital is a specialized center for CCA patients, the number of patients included in this study was limited due to the rarity of the disease as well as due to the fact that PET/CT is not recommended in current guidelines and mostly requested only in cases of ambiguous clinical situations on a case-by-case basis. Despite our encouraging results, further studies with a larger cohort should confirm the clinical impact of ^18^F-FDG-PET/CT in management of CCA patients and may also assess associations with patient outcome.

## Conclusion

Our study suggests that ^18^F-FDG-PET/CT significantly influences clinical management in CCA patients, especially in cases of suspected recurrence based on clinical suspicion and/or inconclusive results of conventional imaging. It supports an accurate staging of the extent of disease and helps to avoid unnecessary imaging and/or invasive procedures in daily routine. Furthermore, it guides decision for reasonable additional tests, for example targeted biopsies. Therefore, a more appropriate and individually tailored treatment may be possible based on the results of PET/CT, which may lead to a prolongation in CCA survival and an improved patient quality of life.
